# A review of the proposed role of neutrophils in rodent amebic liver abscess models

**DOI:** 10.1051/parasite/2016006

**Published:** 2016-02-15

**Authors:** Rafael Campos-Rodríguez, Manuel Gutiérrez-Meza, Rosa Adriana Jarillo-Luna, María Elisa Drago-Serrano, Edgar Abarca-Rojano, Javier Ventura-Juárez, Luz María Cárdenas-Jaramillo, Judith Pacheco-Yepez

**Affiliations:** 1 Sección de Posgrado e Investigación, Escuela Superior de Medicina, Instituto Politécnico Nacional Distrito Federal México; 2 Coordinación de Ciencias Morfológicas, Escuela Superior de Medicina, Instituto Politécnico Nacional Distrito Federal México; 3 Departamento de Sistemas Biológicos, Unidad Xochimilco, Universidad Autónoma Metropolitana Distrito Federal México; 4 Departamento de Morfología, Centro de Ciencias Básicas, Universidad Autónoma de Aguascalientes Aguascalientes México

**Keywords:** Amoebic liver abscess, Rodents, Neutrophil activation, Reactive oxygen and nitrogen species, Peroxynitrite, Neutrophil extracellular traps

## Abstract

Host invasion by *Entamoeba histolytica*, the pathogenic agent of amebiasis, can lead to the development of amebic liver abscess (ALA). Due to the difficulty of exploring host and amebic factors involved in the pathogenesis of ALA in humans, most studies have been conducted with animal models (e.g., mice, gerbils, and hamsters). Histopathological findings reveal that the chronic phase of ALA in humans corresponds to lytic or liquefactive necrosis, whereas in rodent models there is granulomatous inflammation. However, the use of animal models has provided important information on molecules and mechanisms of the host/parasite interaction. Hence, the present review discusses the possible role of neutrophils in the effector immune response in ALA in rodents. Properly activated neutrophils are probably successful in eliminating amebas through oxidative and non-oxidative mechanisms, including neutrophil degranulation, the generation of free radicals (O_2_^−^, H_2_O_2_, HOCl) and peroxynitrite, the activation of NADPH-oxidase and myeloperoxidase (MPO) enzymes, and the formation of neutrophil extracellular traps (NETs). On the other hand, if amebas are not eliminated in the early stages of infection, they trigger a prolonged and exaggerated inflammatory response that apparently causes ALAs. Genetic differences in animals and humans are likely to be key to a successful host immune response.

## Abstract (fr)

L’invasion d’un hôte par *Entamoeba histolytica*, l’agent pathogène de l’amibiase, peut conduire au développement d’un abcès hépatique amibien (AHA). En raison de la difficulté d’explorer les facteurs dépendant de l’hôte et des amibes impliqués dans la pathogenèse de l’AHA chez les humains, la plupart des études ont été menées sur des modèles animaux (par exemple souris, gerbilles et hamsters). Les résultats histopathologiques montrent que la phase chronique de l’AHA chez l’homme correspond à la nécrose lytique ou liquéfiante, tandis que dans les modèles rongeurs on rencontre une inflammation granulomateuse. Cependant, l’utilisation de modèles animaux a fourni des informations importantes sur les molécules et les mécanismes de l’interaction hôte/parasite. Cette synthèse discute donc le rôle possible des neutrophiles dans la réponse immunitaire effectrice pendant l’AHA chez les rongeurs. Les neutrophiles correctement activés réussissent probablement à éliminer les amibes grâce à des mécanismes oxydatifs et non oxydatifs, y compris la dégranulation des neutrophiles, la génération de radicaux libres (O_2_^−^, H_2_O_2_, HOCl) et de peroxynitrite, l’activation des enzymes NADPH-oxydase et myéloperoxydase, et la formation de pièges extracellulaires des neutrophiles. D’autre part, si les amibes ne sont pas éliminées au cours des premiers stades de l’infection, elles déclenchent une réponse inflammatoire prolongée et excessive qui provoque apparemment l’AHA. Les différences génétiques chez les animaux et les humains sont probablement la clé d’une réponse immunitaire réussie de l’hôte.

## 1. Introduction

Because it is very difficult to study the pathogenesis of amebiasis in humans, several animal models have been employed to elucidate pathogenic mechanisms, immune response, and the host-parasite relation. Through the use of these models, important differences have been discovered between rodents and humans in the chronic phase of ALA [124]. The histopathology of the chronic phase of ALA in humans corresponds to lytic or liquefactive necrosis, whereas in rodent models there is granulomatous inflammation [2, 32, 124, 125]. Hepatic damage in hamsters and mice is caused by apoptosis and necrosis rather than the lytic necrosis found in humans in the chronic phase of the disease [14, 112, 126]. However, the information gathered on the pathogenesis of ALA in rodent models has been useful for understanding the effect of different host molecules and mechanisms in humans. The present review discusses the role of neutrophils in the development of ALA based mostly on studies with rodent models.

## 2. The role of host and parasite genotypes in the development of amebiasis

Invasive amebiasis, caused by the enteric pathogen *E. histolytica*, can lead to amebic colitis and ALA. These two disorders are associated with significant levels of morbidity and mortality worldwide [118]. Despite the numerous studies carried out to understand invasive amebiasis and its complications, the mechanisms by which amebas cause host tissue damage have still not been clearly identified. It is evident that both host and parasite genotypes as well as environmental factors (e.g., malnutrition) influence the development of the different forms of the disease (asymptomatic colonization, diarrhea, invasive colitis, liver abscess) [93]. In the host-parasite interaction, neutrophils seem to play a pivotal role in determining resistance or susceptibility.

### 2.1 Genetic differences in susceptibility to *Entamoeba histolytica* infection

Species differ in susceptibility to *Entamoeba histolytica*. Whereas these trophozoites produce liver abscesses in hamsters, gerbils, and some strains of mice, this is not the case in other species of mice or in rats and guinea pigs (even when larger inocula are used in the latter animals). In addition, within the context of a naturally provoked infection, *E. histolytica* only produces invasive lesions in the intestine of humans. Moreover, the majority of people infected with virulent *E. histolytica* do not develop symptomatic disease. This evidence suggests that there are genetic differences between susceptible and resistant species [24].

Indeed, associations have been found between MHC class II alleles and susceptibility [9, 10] or resistance to amebic liver abscess [50]. Also, one study showed an association between protection against intestinal amebiasis and an HLA class II allele or haplotype. However, there is no explanation mechanistically linking HLA class II genes and resistance or susceptibility to invasive amebiasis. It has been proposed that the presence or absence of a particular HLA class II allele could alter the response to amebic infection by changing the repertoire of proteins presented by CD4^+^ T cells [36].

The differences in susceptibility to *E*. *histolytica* between the sexes can also be understood in terms of genetic characteristics. Invasive amebiasis predominantly affects men, which is possibly due to sex-associated hormones or factors linked to the X-chromosome that may act, at least in part, by means of their effects on innate immunity [24]. Studies with animals support this idea. In a murine model, males are more susceptible to ALA development than females [63]. Resistance has been associated with NKT cells activated by the lipopeptidophosphoglycan from *E. histolytica* membranes (EhLPPG), leading to the production of IFN-gamma [64, 65]. Susceptibility in male mice was associated with the production of tumor necrosis factor alpha by monocytes and Kupffer cells [48].

### 2.2 Parasite genotypes also influence infection outcomes

*E. histolytica* strains are highly heterogeneous in relation to pathogenicity. Pathogenic and non-pathogenic strains differ in their ability to cause invasive disease, as well as in several biochemical and molecular criteria [15, 22, 24]. Similarly, the genotypes of *E. histolytica* parasite are significantly different in infected patients without symptoms, with diarrhea/dysentery, or with ALA [5]. Thus, only certain genotypes are capable of causing abscesses, likely due to genetic factors [93].

Many virulence factors of *E. histolytica* have been characterized, including Gal/GalNAc adherence lectin, cysteine proteases (CPs), arginase, amebapores, alcohol dehydrogenase, peroxiredoxin, cyclooxygenase 2, and lipopeptidophosphoglycan (LPPG). One study showed that stable transfectants of virulent *E. histolytica* were strongly inhibited in their cytopathic activity, cytotoxic activity, and ability to induce the formation of amebic liver abscess in hamsters when the expression of the Gal/GalNac lectin 35 kDa subunit was inhibited by antisense RNA. This suggests that the 35 kDa subunit may have a function in amebic pathogenicity [7]. Recently, Matthiesen et al. [70] reported that papain-like cysteine peptidase (CP) genes of *E. histolytica* are differently expressed during ALA formation in distinct rodent models. Non-pathogenic ameba clone A1 that did not induce ALA was transfected, enabling overexpression of CPs that are expressed at high levels during ALA formation. Overexpression of ehcp-b8, -b9, and -c13 restored the pathogenic phenotype of non-pathogenic clone A1, which shows the important role of this molecule in the pathogenicity of *E. histolytica*. Further studies are needed to identify how other specific genes participate in the pathogenesis of amebiasis [69, 93].

## 3. Amebic liver abscess: the possible relation of amebic mechanisms and host inflammation

After entering the host through the digestive tract, amebas arrive in the liver through the blood flow. In this organ, the mechanisms of amebic adherence to immune cells are vital for survival of the pathogen. In the susceptible hamster model of ALA, *E. histolytica* amebas were found in the portal vein of the liver, in the lumen of small branches of the portal vein, and in central veins at 30 min post-inoculation. After 1 h, trophozoites were randomly located in the sinusoids throughout the hepatic lobules [125].

Hence, during the first hour after *E. histolytica* enters the digestive tract, amebic mechanisms (adhesion, cytolysis, and cytopathogenicity) are vital for the trophozoite to be able to survive and proliferate. Of the toxic molecules secreted by amebas, cysteine proteases are particularly important. They are capable of producing a cytotoxic effect on host cells, modulating the cell-mediated immune response (e.g., leukocytes), and carrying out proteolysis of the host extracellular matrix [37, 51, 54, 93, 117, 118]. A question that arises is whether these mechanisms only enable *E. histolytica* trophozoites to survive in a hostile environment, or if they also create ALA. To answer this question, two critical time periods must be considered: the early amebic invasion of the liver (from 1 to 24 h post-infection) and the time of ALA development (3–7 days post-infection).

**Figure 1.**
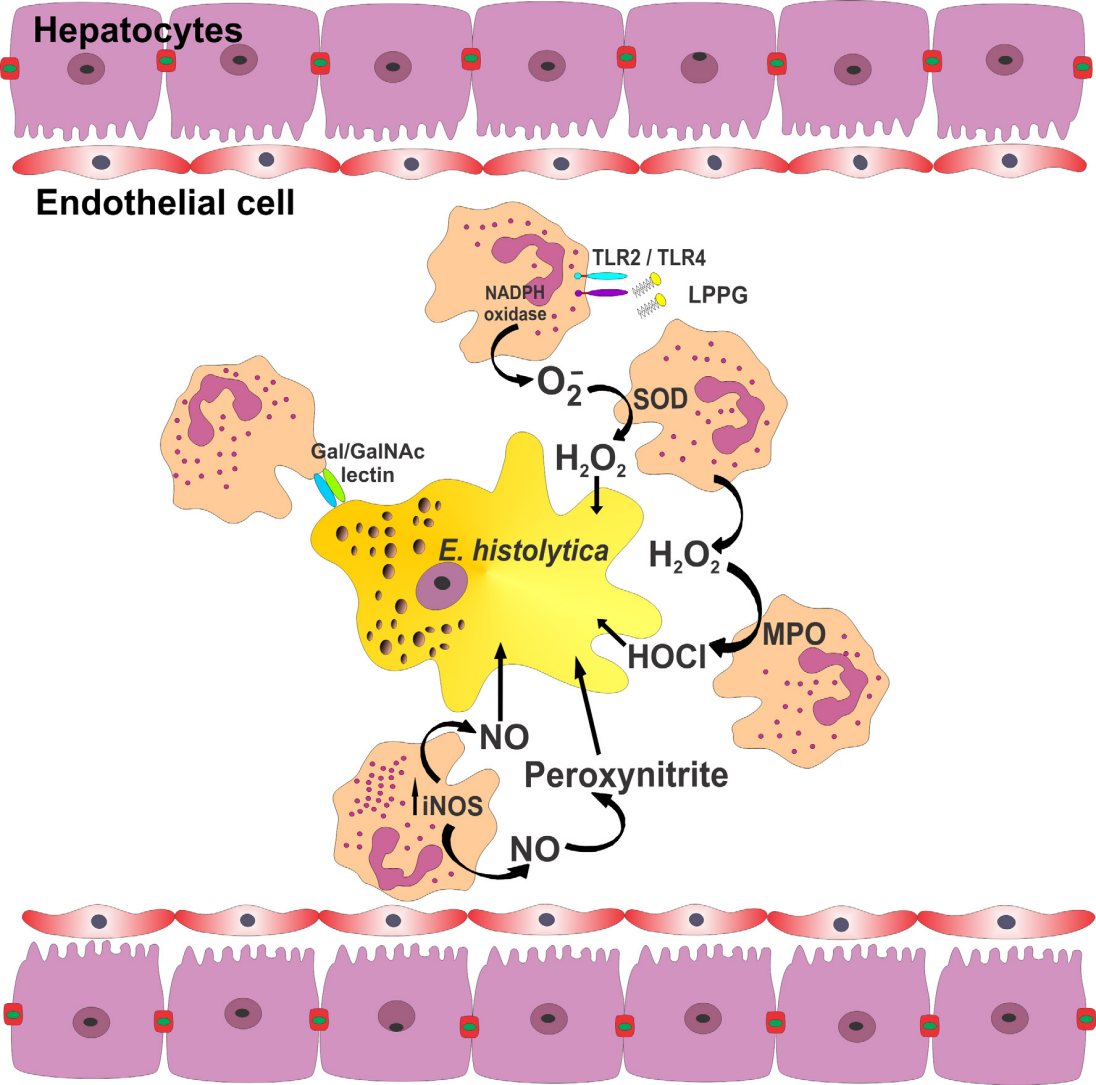
The initial contact between the lectin and neutrophil receptors promotes CR3 expression by neutrophils, and then the interaction of this molecule with iC3b induces neutrophil degranulation. It is likely that neutrophils are activated due to the recognition by TLR-2 and TLR-4 of lipopeptidophosphoglycan (LPPG) from *E. histolytica*. Activated neutrophils undergo an “oxidative burst” that results in an increased production of superoxide (O_2_^−^) by the NADPH-oxidase system. Superoxide dismutase (SOD) rapidly converts O_2_^−^ to hydrogen peroxide (H_2_O_2_, a highly oxidizing agent), and to the substrate of MPO for the formation of hypochlorous acid (HOCl). During this oxidative burst, neutrophils express high levels of iNOS, causing an increase in •NO. Another powerful oxidant is peroxynitrite, formed by the reaction of •NO and O_2_•−.

During an amebic invasion of the liver, phagocytosis and cytopathogenicity can take place in the host liver parenchyma whether or not cysteine proteases are present [52, 79] (Fig. 1). Indeed, during the later stages of ALA, the majority of host cells in contact with amebas appear to be undamaged [25], suggesting that liver tissue damage at this stage is not directly related to amebic mechanisms. In this regard, another study by Olivos-Garcia et al. [80] showed that in the hamster model of amebic invasion of the liver, an immunosuppressor drug, cyclosporine, inhibits tissue damage. Under these conditions, the presence of undamaged parasites was accompanied by few if any leukocytes. Other studies have suggested that the massive lysis of neutrophils by virulent amebas exacerbates host tissue damage in amebic liver abscess [79, 125]. These reports provide evidence that in the acute and chronic phases of ALA, host tissue damage is caused more by the immune response than by amebic mechanisms.

In a gerbil model of ALA, early lesions were accompanied by an acute inflammatory response, mainly constituted by a large number of neutrophils and eosinophils. There was widespread lysis of these immune cells accompanied by the proliferation of ALA. The authors asserted that in the gerbil model, the fact that the acute inflammatory response is inefficient in destroying trophozoites allows for the development of ALA, a process that is aggravated by the lytic secretory products of both inflammatory cells and necrotic areas [27].

Since liver tissue damage during amebiasis is likely to be a downstream event in regard to amebic virulence factors, the host immune response must be explored to determine the mechanisms of ALA. It is known that both the innate and adaptive immune responses can contribute to the protection of the host during amebic invasion. Interestingly, several studies with animal models have demonstrated that virulent amebas can be eliminated within a few hours of infection, which could only be explained by the innate response [53, 109, 128].

Shibayama injected inflammatory substances (incomplete Freund’s adjuvant and mineral oil) into the hamster peritoneum, and animals were then challenged with *E. histolytica* trophozoites, demonstrating protection against the development of ALA [113]. The authors concluded that non-specific stimulation of peritoneal exudate cells, mainly constituted by neutrophils and macrophages, prevented amebic invasion of the liver.

Hence, it has been proposed that resistance to amebic invasion is the result of an efficient innate immune response [53, 113, 128] and that susceptibility is a consequence of the inadequacy of the same [25, 117, 125]. Regarding susceptibility, abundant evidence supports the idea that a chronic inflammatory response is one of the principal causes of host tissue damage [24, 25, 113, 131]. Indeed, virulent *E. histolytica* trophozoites cause little or no liver tissue damage in hamsters in the absence of inflammatory cells [79, 90].

For example, leukopenic hamsters with no inflammatory cells (after whole body radiation at 800 rads) were infected with amebas, which rapidly disappeared from the liver without producing any lesions [79]. Likewise, the lack of an inflammatory response to amebas in the hamster liver results in the early disappearance of *E. histolytica* trophozoites from the parenchyma and no liver damage [90].

In summary, inflammation appears to be pivotal and amebic molecules dispensable for the formation of liver abscesses during amebiasis. Since neutrophils are the first elements of the inflammatory infiltrate to arrive at sites of amebic presence, as well as the most numerous cells in the early stages of the inflammatory response (1–24 h post-inoculation), the aim of the current review was to explore the mechanisms of activated neutrophils that may contribute to the elimination of *E. histolytica*.

## 4. Anti-amebic activity of neutrophils

Neutrophils have long been recognized as the most important killers of invasive microorganisms in the body [6, 77, 85, 108, 115, 116, 122]. They are not only the most abundant cells at inflammatory sites, but also the first to interact with invasive amebas [27, 53, 125]. In animal models of amebic liver abscess, neutrophils have been found in the liver from 30 min to a few hours after inoculation with *E. histolytica* [27, 53, 123, 125]. Moreover, neutrophils have proven to be effective killers of ameba. An *in vitro* study compared activated and unactivated (with rIFN-γ and rTNF-α) human neutrophils, finding that the former show a 97% increase in amebic killing. Neutrophils enhance their amebicidal activity after cytokine treatments [35].

In a study in which amebas were inoculated in Balb/c and C3H/HeJ mice (resistant animal models of ALA), neutrophils and other immune cells were able to limit the amebic lesion by the fourth day [53]. The absence of neutrophils in another *in vivo* study pointed to the important role of these immune cells in controlling the size of ALA. With a severe combined immunodeficient (SCID) mouse model [109], animals were neutrophil-depleted with monoclonal anti-neutrophil antibodies RB68C5. After infection with *E. histolytica* trophozoites, these neutrophil-depleted mice had larger amebic liver abscesses at early stages than control animals (without depleted neutrophils). Histological analysis showed that neutrophil-depleted mice presented an absence of inflammatory cells surrounding the necrotic area. This study supports the idea that neutrophils play a key role in controlling amebic liver abscess in SCID mice. Another study corroborated the same idea [128], reporting significantly larger ALAs in neutrophil-depleted mice. Neutrophil activation can set in motion various mechanisms of the inflammatory response, including the activation of other immune components. One of these components is mononuclear cells, which are apparently the principal actors that combat abscess formation during the chronic stage of ALA.

Although neutrophils seem to be important for eliminating invading amebas, they can also apparently contribute to host tissue damage. This seemingly double-edged role of neutrophils, evidenced by various studies, seems to depend on the time frame of an amebic invasion. One such study reported that after polymorphonuclear leukocytes surrounded amebas in the liver, the lysis of liver parenchymal tissue continued [125], evidently not the result of amebic mechanisms. Other leukocytes underwent massive destruction, which favored greater necrosis, hemorrhaging of parenchymal tissue, and the formation of ischemic areas [79, 125].

A study carried out with C57BL76 mice reported that neutrophils were the first and majority of immune cells to infiltrate the liver on day 1 of an amebic invasion [48]. In the chronic stage of amebiasis (3–7 days post-inoculation), the diffuseness of neutrophil staining suggested neutrophil cell death. At this time, the inflammatory infiltrate was represented principally by macrophages. The authors then depleted neutrophils from another set of mice by using anti-Ly6G or anti-GR1antibodies, and inoculated the animals with *E. histolytica*. Anti-Ly6G recognizes the neutrophil-specific cell surface molecule Ly6G and selectively depletes neutrophils. Compared with wild type mice, neutrophil depletion with anti-Ly6G led to a slight but not significant reduction in the abscess size. The authors also used anti-GR1, which recognizes Ly6C. This receptor is expressed on both neutrophils and monocytes. Depletion of immune cells with the anti-GR1 antibody caused a significant decrease in ALA size on day 3 post-inoculation.

The authors concluded that neutrophils do not have a beneficial or protective role in ALA, but in fact contribute to liver damage. We would argue that the effect of the Ly6G or anti-GR1 antibodies should have been measured from 1 to 24 h post-inoculation, the time period that neutrophils probably have their most effective action during an amebic infection. The fact that they found evidence of neutrophils contributing to liver damage at day 3 post-inoculation is in agreement with the processes we have described corresponding to the chronic phase of ALA. The formation of the abscesses is accompanied by the massive lysing of neutrophils.

On the other hand, the same authors [48] concluded that Ly6C+ mononuclear cells but not Ly6G+ neutrophils are critical cell mediators of tissue destruction during ALA formation. However, the anti-GR1 antibody depletes both neutrophils and monocytes. Therefore, the decrease in ALA size could be attributed to the additive effect of both these immune cell types. Moreover, the decrease in the size of ALA after pretreatment with the anti-GR1 antibody is to be expected, as it leads to a decrease in monocytes, which are the principal immune molecules participating in the inflammatory response during the stages of chronic inflammation on day 3.

## 5. Neutrophil effector mechanisms

It seems that adequate or inadequate activation of neutrophils is one of the key factors in determining resistance or susceptibility to *E. histolytica*, an idea supported by many studies [25, 53, 88, 90, 125, 128]. Indeed, it has been shown that neutrophils are either activated or inactivated upon interacting with trophozoites and their molecules [35, 44, 46].

Lipophosphoglycan-like (LPG) and lipopeptidophosphoglycan (LPPG) glycosylphosphatidylinositol-linked molecules of *E. histolytica* probably behave like pathogen-associated molecular patterns (PAMPs) that are recognized by TLR-2 and TLR-4, thus leading to the activation of neutrophils. This is known to occur with human monocytes and dendritic cells [66, 67, 131].

**Figure 2.**
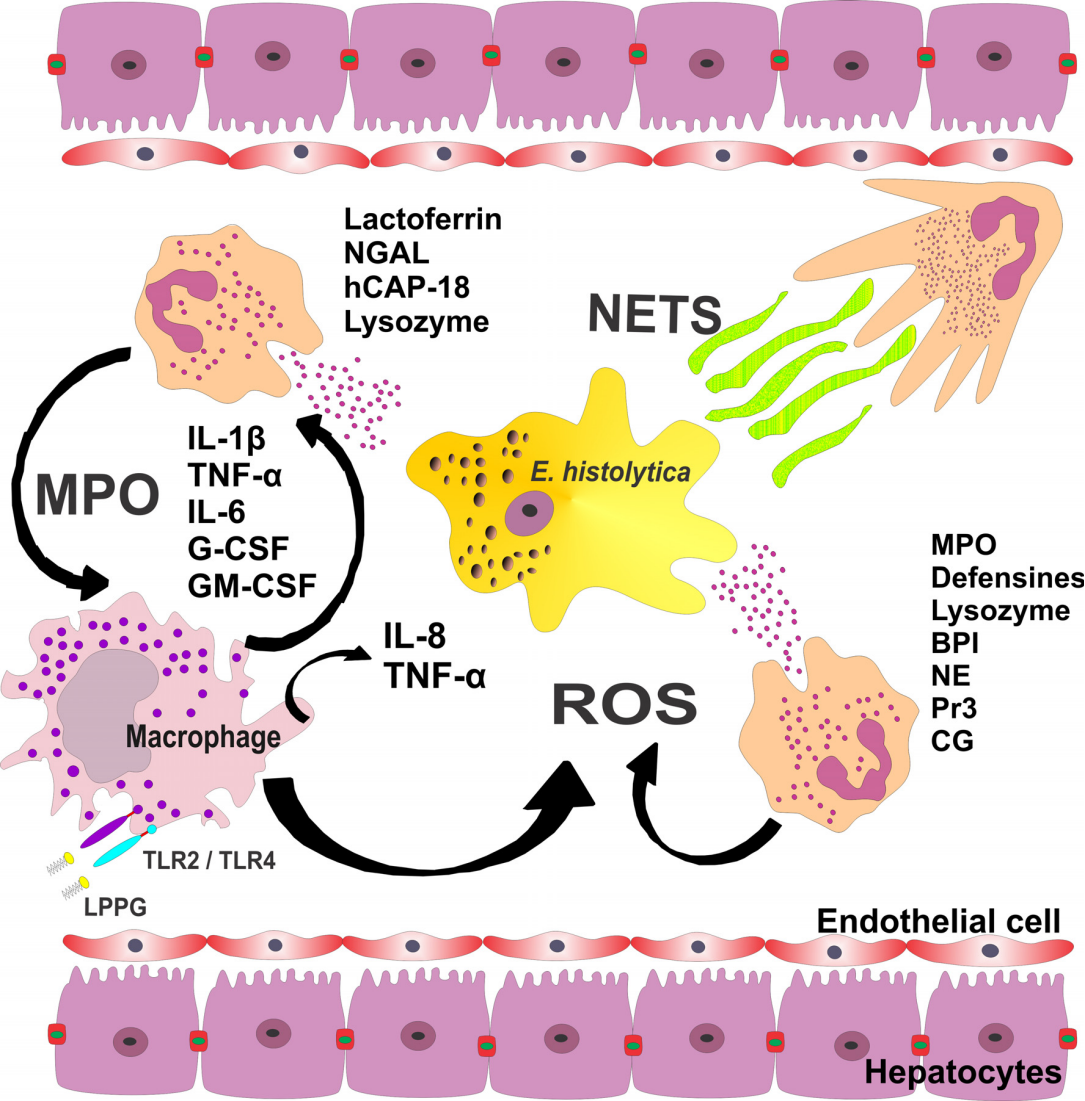
Activated neutrophils provide signals for the activation and maturation of macrophages, which in turn release IL-1β, TNF-α, G-CSF, and GM-CSF. These cytokines extend the life span of neutrophils at sites of inflammation. The interaction of lipopeptidophosphoglycan (LPPG) with TLR-2 and TLR-4 results in the activation of NF-kappa B and the release of IL-8, IL-10, IL-12p40, and TNF-α from human macrophages. Activated neutrophils enhance the production of reactive oxygen species (ROS), activating NF-kB and increasing neutrophil degranulation. Primary granules contain MPO, defensins, lysozyme, bactericidal/permeability-increasing protein (BPI), neutrophil elastase (NE), proteinase 3 (PR3), and cathepsin G (CG). Secondary granules are characterized by the presence of lactoferrin, neutrophil gelatinase-associated lipocalin (NGAL), human cationic antimicrobial protein 18 or cathelin (hCAP-18), and lysozyme. MPO can bind to monocytes, which might lead to the production of ROS and proinflammatory cytokines. Another function of neutrophils is the formation of neutrophil extracellular traps (NETs), composed of DNA bound with antimicrobial components (e.g., bacterial permeability-increasing protein, myeloperoxidase, elastase, lactoferrin). NET formation may have an important role in combating amebas.

Activated neutrophils can eliminate non-pathogenic amebas in individuals who are susceptible to pathogenic strains [46, 75, 117, 119], and they produce cytokines that could influence or determine the evolution of the immune response against diverse pathogens including *E. histolytica* [53]. These cytokines have a variety of functions, such as stimulating the production of reactive oxygen species (ROS), activating NF-kB, and increasing neutrophil degranulation [26] (Fig. 2).

It is likely that activated neutrophils are attracted to sites of amebic invasion by various phenomena, including the chemotactic activity of amebic membrane proteins [28, 44, 58, 105] as well as C5a and C3a fragments produced by activation of the complement on the amebic surface [46, 98, 99]. Neutrophils also likely follow the trail of chemokines secreted by cells exposed to *E. histolytica* [46].

These immune cells make close contact with pathogenic and non-pathogenic strains of *E*. *histolytica*, showing higher chemotaxis *in vitro* toward live pathogenic amebas [105]. The interaction between the 260 kDa Gal/GalNAc lectin of *E. histolytica* and neutrophil receptors may initially mediate the ameba-host encounter [23, 31, 97], possibly leading to greater expression of CR3 molecules and thus the formation of neutrophil conglomerates around the ameba by homotypical recognition among molecules.

Whatever the exact mechanism may be, the formation of neutrophil conglomerates is probably pivotal to the amebicidal activity of the immune response. In one study [53], Balb/c and C3H/HeJ mouse strains (resistant animal models) were inoculated with *E. histolytica* to analyze the cellular immune response in these animals. The results show that amebas were surrounded by neutrophils containing nitric oxide synthase (iNOS) as well as by macrophage leukocytes. Neutrophils, macrophages, and other immune cells limited the amebic lesion and eliminated the parasites by the fourth day [53]. It has also been found that trophozoites are surrounded by neutrophils in hamsters and gerbils (susceptible animal models) [27, 125].

Several studies from ALA models have shown that the aggregation of inflammatory cells, among them neutrophils, is the first line of defense against amebas in ALA [63, 125, 129]. Once neutrophils aggregate with other inflammatory cells, they form conglomerates that surround amebas. Under these conditions, it is known that neutrophils attack amebas by the release of oxidative molecules (superoxide, peroxide, NO, and peroxynitrite) as well as with non-oxidative mechanisms (degranulation, release of MPO) [35, 83, 109, 128]. Apart from their direct role in surrounding and attacking amebas, neutrophils also influence the function of other immune cells. On the other hand, one mechanism that can be discarded in regard to *E. histolytica* is phagocytosis, because neutrophils (with a diameter of 12–15 μm) cannot phagocyte amebas (with a diameter of 20–40 μm).

### 5.1 Oxidative mechanisms

#### 5.1.1 Superoxide and peroxide

Activated neutrophils undergo an “oxidative burst”, during which time the large NADPH-oxidase complex assembles at the phagosomal and plasma membranes and transfers electrons to molecular oxygen, yielding superoxide (O_2_^−^). Through catalysis induced by superoxide dismutase (SOD), O_2_^−^ is rapidly converted to hydrogen peroxide (H_2_O_2_). This molecule is both a highly oxidizing agent and the substrate of MPO. The latter molecule transforms H_2_O_2_ into hypochlorous acid (HOCl), which is the most bactericidal oxidant of neutrophils [47, 71, 77, 107, 122, 127] (Fig. 1).

It is possible that large quantities of O_2_^−^ and H_2_O_2_ are produced during the ameba-neutrophil interaction (Fig. 1). Several studies have indicated that H_2_O_2_ effectively kills *Entamoeba*. However, virulent *E. histolytica* is more resistant to H_2_O_2_ than non-virulent strains and species such as *E. histolytica* Rahman [30, 41, 42, 76, 95].

Inflammatory cells reportedly release reactive oxygen species (ROS) and reactive nitrogen species (RNS) that kill pathogens [107]. However, PMNs are able to fulfill their function with both normal and defective production of ROS [74], which casts a doubt on whether these free radicals contribute directly and significantly to amebic killing *in vivo*. It is possible that ROS are only activators of granule proteins or other effector molecules.

The generally accepted view is that the system of NADPH oxidase promotes microbial killing through the generation of ROS and the activity of MPO. An alternative concept is that the main function of NADPH oxidase is to drive ion fluxes across the vacuolar membrane and to adjust the pH and ionic composition within the vacuole. In the case of amebiasis, either of these conditions would optimize the killing of amebas and regulate the digestive function of enzymes (e.g., elastase and cathepsin G) that could be released into the vacuole from the cytoplasmic granules [100, 107, 108]. In this way, the largely cationic microbicidal enzymes released would be made soluble by dissociation from the sulfated negatively charged granule matrix in an environment with a pH favorable for optimal activity [121]. In brief, it is possible that the predominant function of NADPH oxidase is to optimize conditions for the efficient function of the granule enzymes in the phagocytic vacuole (Fig. 2).

#### 5.1.2 Nitric oxide

It has been demonstrated that NO is involved in amebicidal mechanisms of neutrophils and macrophages. For example, nitric oxide synthase (NOS) activity has been reported to be an important molecule for the control of hepatic amebiasis in immunocompetent mice [53, 110]. Neutrophils have high expression of iNOS, especially when in contact with amebas. However, this expression is less than that of macrophages. On the other hand, the abundance of neutrophils in acutely inflamed tissue [53] may compensate for this lower expression.

Several studies have explored the role of iNOS and NO in the immune response to *E. histolytica*. For example, severe combined immunodeficiency (SCID) mice and iNOS knockout mice were employed to determine the *in vivo* role of IFNγ and NO in host defense against amebic liver abscess formation [110]. SCID mice were genetically engineered to assure the absence of the IFNγ receptor chain. The role of NO was assessed with knockout mice, in which the gene coding for iNOS was disrupted. When inoculated with amebas, the SCID and knockout mice developed larger ALAs than SCID control mice, showing that IFNγ and iNOS play a role in innate immune protection against the development of ALA, probably by stimulating NO production in neutrophils. Although the stimulus that induces iNOS expression in neutrophils is still unknown, it could be similar to that described in macrophages, where the interaction of bone marrow monocytes with the Gal/GalNAc lectin of *E. histolytica* induces the synthesis of TNF. This cytokine and TGF-β1 stimulate IFNγ-primed macrophages to produce NO [62].

Nevertheless, it is likely that the role of NO in combating amebas is not direct. Even though in the serum of hamsters (susceptible model) iNOS mRNA expression and NO levels are greater with liver abscesses than in healthy animals [82, 94], the amebas continue to survive and proliferate (Fig. 1). Curiously, hepatic tissue damage was found to be reduced or prevented with the administration of nitric oxide synthase inhibitors [82]. Regarding the high concentrations of NO that exist during the development of ALA, trophozoite resistance to this molecule has been evidenced *in vitro* [96] and *in vivo* [82, 94] (see Sect. 6).

*E. histolytica* can probably resist the destructive action of NO and ROS because these trophozoites express high levels of antioxidant proteins, such as peroxiredoxin, flavoprotein A, superoxide dismutase, and rubrerythrin [18, 20, 21, 25, 30, 68]. Moreover, the blood flow to necrotic areas is reduced or blocked. Thus, at these sites the scarce supply of oxygen results in lower concentrations of NO and ROS.

#### 5.1.3 Peroxynitrite

It is generally accepted that the system of NADPH oxidase and H_2_O_2_ promotes microbial killing through the activity of myeloperoxidase. In a similar manner, NO may promote microbial killing mainly through the formation of peroxynitrite [84]. Although not a free radical, peroxynitrite is a powerful oxidant. The peroxynitrite anion (ONOO−), a short-lived oxidant species, is produced by the reaction of the nitric oxide (•NO) and superoxide (O2•−) radicals at diffusion-controlled rates [120]. NO is produced by NOS and O_2_ by NADPH oxidase. Therefore, the synthesis of peroxynitrite occurs in places where both these substrates are found, such as phagocytes (neutrophils, monocytes, macrophages, dendritic cells, mast cells) and activated endothelial cells [120].

To be able to survive and provoke an uncontrolled inflammatory response, trophozoites would require a better defense system against peroxynitrite than that of the susceptible host. There are two general mechanisms through which *E. histolytica* could protect itself against peroxynitrite: prevention (inhibition and scavenging of precursors) and interception (decomposition of peroxynitrite into non-toxic molecules) [11, 57].

### 5.2 Non-oxidative mechanisms

#### 5.2.1 Granules

It is not known whether ROS contribute directly to amebic killing *in vivo* or are only activators of granule proteins, which are oxygen-independent effectors located in four types of granules in neutrophils: primary (azurophilic), secondary (specific), tertiary (gelatinase), and secretory vesicles. All of these granules contain a surplus of peptides and proteins that directly or indirectly kill microbes.

Azurophilic granules (peroxidase-positive or primary granules) contain myeloperoxidase (MPO), defensins, lysozyme, bactericidal/permeability-increasing protein (BPI), and a number of serine proteases, including neutrophil elastase (NE), proteinase 3 (PR3), and cathepsin G (CG). These granules function as the primary repository for the molecular weaponry of neutrophils. Secondary granules are characterized by the presence of a glycoprotein (lactoferrin) and several antimicrobial compounds, including neutrophil gelatinase-associated lipocalin (NGAL), human cationic antimicrobial protein 18 or cathelin (hCAP-18), and lysozyme [61]. Hence, through enzymes and cationic antimicrobial peptides, neutrophils could participate in amebic destruction as well as in the recruitment of cells that participate in both the innate and the specific immune responses [29] (Fig. 2).

Tertiary granules contain few antimicrobials, but they serve as a storage location for a number of metalloproteases, such as gelatinase and leukolysin. Secretory vesicles, also commonly considered part of the neutrophil granule, contain predominantly plasma-derived proteins such as albumin. The membrane of secretory vesicles serves as a reservoir for a number of important membrane-bound molecules employed during neutrophil migration [6, 16, 71, 77, 127].

Granules contain three main types of antimicrobials: (a) cationic peptides and proteins that bind to microbial membranes, (b) enzymes, and (c) proteins that deprive microorganisms of essential nutrients. At the inflammatory site, granules in the activated neutrophils are mobilized and fuse with either the phagosome or the plasma membrane, releasing their potent antimicrobials into the affected tissue [6, 77].

Little is known about the participation of these antimicrobial granular components in the destruction of amebas. They perhaps make contact with amebas through the extracellular degranulation of neutrophils and/or the lysis of neutrophils by *E. histolytica*. However, most granular components require activation by enzymes (serine proteases: cathepsin, elastase, and proteinase 3) that are not present in inflamed tissue [12, 107]. Therefore, one granular component – MPO, an enzyme that works extracellularly – could be of particular importance.

#### 5.2.2 Myeloperoxidase

Although neutrophils are too small to phagocytize *E. histolytica*, there is abundant evidence that they do indeed surround these pathogenic invaders [27, 33, 53, 88, 90, 125]. In this walled-off space, neutrophils could release HOCl, an amebicidal molecule produced by the MPO enzyme.

MPO is a cationic enzyme located in the primary azurophilic granules of neutrophils and immature monocytes [47, 55, 56]. When released by neutrophils, MPO binds to monocytes that express mannose receptors [111], which might lead to the production of reactive oxygen species (ROS) and proinflammatory cytokines (i.e., TNF-α, IL-1, IL-6, IL-8, and GM-CSF) [59]. Whereas inactive MPO is capable of inducing great quantities of pro-inflammatory cytokines, active MPO is more efficient for ROS production [40, 43, 60] (Fig. 2).

MPO uses the H_2_O_2_ produced by neutrophils to oxidize chloride ions and produce the highly cytotoxic HOCl (see Sect. 4.1.1). For the same purpose, it may use the SOD and NADPH flavin oxidoreductase produced by trophozoites, as occurs in other illnesses [47, 56, 71, 77, 91, 107, 127]. One study showed that hamster MPO binds to the surface of *E. histolytica in vitro*, causing important morphological and ultrastructural alterations and culminating in complete amebic destruction [83]. Thus, HOCl can in fact damage and kill trophozoites, indicating that MPO could indeed play an essential role in the innate immune response against *E. histolytica* [47, 71, 107]. Apart from provoking alterations in nuclear morphology and disturbances in the plasma membrane, MPO causes an increase in the number of vacuoles in the cytoplasm and a decrease in the presence of glycogen. Through its oxidative activity, HOCl produced by MPO modifies proteins, lipids, and DNA, and distorts the intracellular redox balance by depleting physiological antioxidants, such as ascorbate and glutathione [56].

A very recent study in our laboratory evaluated the behavior of MPO in Balb/c mice (resistant model) exposed to hepatic amebiasis. The MPO protein was observed in neutrophils present in the hepatic parenchyma, especially in inflammatory foci with damaged *Entamoeba histolytica* trophozoites. Moreover, the *in situ* enzymatic activity of MPO increased significantly as the time of infection elapsed. Based on these results, we suggest that this enzyme could destroy amebas and therefore play an important role in the resistance mechanisms of Balb/c mice against hepatic infection (unpublished data).

Based on the evidence presented here, we propose a model of the amebicidal effect of MPO. In the first stage of the amebic invasion, *E. histolytica* trophozoites spread through the bloodstream to the liver and are exposed to a high oxygen partial pressure and to circulating neutrophils [3, 46, 106]. Neutrophils and other immune cells then rapidly accumulate around the trophozoites [53, 125] and release HOCl (produced by MPO and H_2_O_2_) thus inducing toxicity. Moreover, it is possible that MPO is released into the extracellular fluid by leakage during cell lysis or by the exposure of neutrophils to a variety of soluble stimuli [55] and that it could be more efficient in the area of contact between immune cells and the amebic membrane. The activity of the MPO–H_2_O_2_–HOCl system seems to be an important mechanism by which the innate immune response prevents the invasion of *E. histolytica* [83, 113].

#### 5.2.3 Neutrophil extracellular traps (NETs)

In addition to phagocytizing bacteria, secreting cytotoxic molecules, and affecting other immune molecules, another function of neutrophils was identified in 2004: the formation of NETs [71]. Due to intense efforts made since then, it is now known that NETs have a microbicidal function and possibly form a physical barrier that avoids the further spread of bacteria [6, 17, 49, 85, 107, 122, 130]. Composed of DNA bound with antimicrobial components (e.g., bacterial permeability-increasing protein, myeloperoxidase, elastase, and lactoferrin) [49, 122], these networks of extracellular fibers are able to capture gram-positive and gram-negative bacteria [130].

It has been shown that NETs released from neutrophils can trap parasites such as *Leishmania* sp., *Plasmodium falciparum*, and *Toxoplasma gondii* [1, 13, 39, 45, 49]. Although the molecular mechanism of NET formation is still unclear, it is known that MPO, NADPH oxidase, and elastase are required [38, 49, 73, 85–87, 122]. Some pathogens that are too large to be phagocytized, such as fungal hyphae, are trapped by NETs [6, 127]. This may also be the case for helminths and amebas (Fig. 2). Although NET formation had not previously been described in the literature as a response to *E. histolytica*, recent work in our laboratory has shown that the release of these structures participates in the interactions between human PMN cells and *E. histolytica* trophozoites, and that MPO is present in them (unpublished work).

## 6. Defense mechanisms of amebas

The capacity of *E. histolytica* to provoke ALA has been considered for many decades to be primarily related to amebic mechanisms (adhesion molecules, proteases, membrane proteins, and amebapores). Cysteine proteases (especially those located on the surface of *E. histolytica*) can destroy neutrophils and macrophages and degrade the granular proteins of neutrophils (Fig. 1). However, several findings suggest that cysteine proteinases either are not involved or play a minor role in tissue damage [25, 92].

The amebic secretion of serine protease inhibitors (serpins) seems to be another defense mechanism of *E. histolytica*. These molecules inhibit neutrophil serine proteases (e.g., proteinase 3, cathepsin, and elastase, known collectively as serprocidins) and there is evidence of their activity in *E. histolytica*. It is known that the sequenced *Entamoeba* genome contains serpin genes [102] and that serpins located in the cytoplasm of *E. histolytica* are secreted in the presence of specific mammalian cells. It has been reported that serpin alpha 1-antitrypsin inactivates neutrophil elastase (NE) when it is no longer necessary [6] and that one serpin of *E. histolytica* – cathepsin G – inactivates a serine proteinase secreted by human neutrophils [101].

Additionally, several enzymes of *E. histolytica* can inhibit the oxidative response of neutrophils [4, 8, 104]. For instance, neutrophils have an iron-containing superoxide dismutase [18, 19] and a bifunctional NADPH flavin oxidoreductase [21] that transform superoxide anions to H_2_O_2_. However, this molecule can be reduced and detoxified by a surface peroxiredoxin of *E. histolytica* [20, 30, 34]. Moreover, virulent amebas exposed to great concentrations of O_2_ are able to reduce these molecules as well as H_2_O_2_ by the reactivation of a reversible pyruvate, the ferredoxin oxidoreductase (PFOR) enzyme, and therefore show high resistance to increased levels of H_2_O_2_ [95].

As previously mentioned (see Sects. 5.1.2 and 5.1.3), virulent *E. histolytica* trophozoites can probably resist the destructive action of NO, ROS, and peroxynitrite. The uniformity and low negative charge of the amebic surface coat are also an essential amebic defense mechanism. It can be appreciated that several self-defense mechanisms of amebas are apparently involved in a survival function, which if successful, triggers a prolonged inflammatory response by the host.

## 7. Inflammation and hypoxia

A host invasion by *E. histolytica* trophozoites provokes inflammation, which leads to vasodilation and induces hypoxia and ischemia in the early and late stages of amebic liver abscesses in susceptible (hamster) or resistant (mouse) species. Hypoxia (low oxygen) or anoxia (complete lack of oxygen), acidosis (high H+ concentration), and abundant free oxygen radicals are characteristic features of inflamed tissues. Hence, the battle between neutrophils and pathogenic microorganisms, including *Entamoeba*, takes place mainly in hypoxic microenvironments [78].

Hypoxia is caused by decreased perfusion, increased interstitial pressure, microvascular injury, thrombosis, and the occlusion of blood vessel by amebas, coupled with the local consumption of O_2_ by parasites and recruited inflammatory cells [79, 81, 89, 123–125]. Thus, at inflammatory sites, ischemia contributes to tissue damage and decreases the exposure of trophozoites to serum components (especially the complement).

The relatively anaerobic microenvironment created is favorable for parasites [81]. However, it is also favorable for human neutrophils, because hypoxic conditions inhibit their rate of constitutive apoptosis [72] that could otherwise be triggered by amebic adherence and oxidation (see Sect. 1.1). For example, it has been reported that ROS-dependent neutrophil apoptosis is probably hampered when the neutrophils encounter *E. histolytica* in inflamed tissue *in vivo* [114], which could explain why neutrophils close to amebas remain viable, while those farther away (outside of the inflammatory focus) do not [125].

Hence, if neutrophils and other immune molecules cannot get rid of the host of *E. histolytica* during the early stages of amebic invasion, the massive lysing of neutrophils (outside of the inflammatory foci) by amebas apparently helps tip the balance in favor of the latter during the battle between the invader and its host. In an *in vitro* study [103], the interaction of neutrophils and amebas in the presence of Chang liver cells showed that the lysed neutrophils enhanced the destruction of the liver cell monolayer in a concentration-dependent manner. The authors concluded that the *in vitro* lysis of human neutrophils by *E. histolytica* enhances the destruction of liver tissue, probably through the release of neutrophil oxidative products.

## 8. Conclusions

Increasing evidence strongly suggests that liver abscesses are formed during amebiasis by mechanisms of host inflammatory response. Prolonged and exaggerated inflammation is triggered by amebas that are capable of surviving in the host environment. Hence, it seems that adequate host innate immune response, which would eliminate amebas and prevent chronic inflammation, is the key to avoiding ALA.

In the early stages of invasion, *E. histolytica* amebas are mainly surrounded by neutrophils and other cells of the host innate immune response. If properly activated, these immune cells are probably successful in eliminating amebas through oxidative and non-oxidative mechanisms, including neutrophil degranulation, the generation of free radicals (O_2_^−^, H_2_O_2_, HOCl) and peroxynitrite, the activation of NADPH-oxidase and MPO enzymes, and the formation of NETs. Neutrophils are also involved in the regulation of other cells of the innate and adaptive immune responses. The battle between the invader and the host takes place within the hypoxic environment of inflammatory sites, which has favorable effects on both amebic and neutrophil function. Future studies are needed to deepen the understanding of the molecules and mechanisms involved in the pathogenesis of ALA in rodents and humans.

## Conflict of interest

The authors declare that there is no conflict of interest regarding the publication of this paper.
